# Supplementary material for: Foodborne Pathogen Dynamics in Meat and Meat Analogues Analysed Using Traditional Microbiology and Metagenomic Sequencing
Source: Antibiotics (Basel). 2023 Dec 21;13(1):16. doi: 10.3390/antibiotics13010016 (PMC10812575; doi:10.3390/antibiotics13010016)
Supplement: Supplementary file 1 [file antibiotics-13-00016-s001.zip › antibiotics-2768517-supplementary.pdf]

Supplementary Information for

Foodborne pathogen dynamics in meat and meat analogue analysed by traditional microbiology and metagenomic sequencing

Supplementary Table S1. Equations to determine cfu/mL from optical density at 600nm.

Bacteria	Equation to determine colony-forming units
Listeria monocytogenes	Y = (12.98 x 10^8) X - 1.71 x 10^6
Salmonella Typhi	Y = (6.895 x 10^8) X - 0.319 x 10^6
Escherichia coli 0157:H7	Y = (5.841 x 10^8) X - 5.04 x 10^6

Y = Colony forming units; X = Optical density at 600nm.

Supplementary Table S2. Optical density at 600nm and corresponding colony forming units used for spiking in pig, pea, and soy juice.

Microorganisms	OD	CFU/mL
Listeria monocytogenes	0,209	2,6 x 10^8
Salmonella Typhi	0,588	4,05 x 10^8
Escherichia coli HEHA16	0,724	4,1 x 10^8
Cronobacter sakazakii	0,461	2,6 x 10^8

OD = Optical density at 600 nm.

Supplementary Table S3. Optical density at 600nm and corresponding colony forming units used for spiking in beef and chicken juice.

Microorganisms	OD	CFU/mL
Listeria monocytogenes	0,209	2,6 x 10^8
Salmonella Typhi	0,604	4,1 x 10^8
Escherichia coli HEHA16	0,538	3,1 x 10^8
Cronobacter sakazakii	0,318	1,8 x 10^8

OD = Optical density at 600 nm.

Supplementary Table S4. Bacterial concentrations expressed as cfu/100 μL in the 5 mL meat/meat analogue juices.

Food matrix	*Escherichia coli* HEHA16	*Listeria monocytogenes*	*Salmonella* Typhi	*Cronobacter sakazakii*
PIG	4,1 x 10^6	2,6 x 10^6	4,05 x 10^6	2,6 x 10^6
PEA	4,1 x 10^6	2,6 x 10^6	4,05 x 10^6	2,6 x 10^6
SOY	4,1 x 10^6	2,6 x 10^6	4,05 x 10^6	2,6 x 10^6
CHICKEN	3,1 x 10^6	2,6 x 10^6	4,1 x 10^6	2,6 x 10^6
BEEF	3,1 x 10^6	2,6 x 10^6	4,1 x 10^6	2,6 x 10^6

Supplementary Table S5. Relative abundances of the detected pathogenic bacterial taxa using metagenomic sequencing in all included meat and meat analogues where a single bacterium was inoculated into the matrix and incubated over time. 1, 2 and 3 represent the 3 days of incubation, day 1, day 2 and day 3.

C. sakazakii		*E. coli*		*L. monocytogenes*		*S.* Typhi	
Beef Crono 1	89,14434	Beef Coli 1	90,67842	Beef List 1	92,0036	Beef Salmo 1	82,51322
Beef Crono 2	88,86935	Beef Coli 2	87,20641	Beef List 2	93,38248	Beef Salmo 2	79,89853
Beef Crono 3	89,15337	Beef Coli 3	86,54829	Beef List 3	93,38008	Beef Salmo 3	79,06574
Chicken Crono 1	86,75402	Chicken Coli 1	13,06994	Chicken List 1	75,53367	Chicken Salmo 1	77,8446
Chicken Crono 2	88,61094	Chicken Coli 2	6,24416	Chicken List 2	73,94991	Chicken Salmo 2	78,99075
Chicken Crono 3	87,65216	Chicken Coli 3	26,41271	Chicken List 3	83,52631	Chicken Salmo 3	78,215
Peas Crono 1	89,80104	Peas Coli 1	84,51989	Pig List 1	78,01425	Peas Salmo 1	90,04712
Peas Crono 2	89,77533	Peas Coli 2	86,75854	Pig List 2	92,48886	Peas Salmo 2	90,58372
Peas Crono 3	89,66048	Peas Coli 3	86,49998	Pig List 3	93,01339	Peas Salmo 3	90,49974
Pig Crono 1	87,15304	Pig Coli 1	82,71754	Soy List 1	8,67433	Pig Salmo 1	90,03666
Pig Crono 2	88,81244	Pig Coli 2	81,1363	Soy List 2	DNA was too fragmented	Pig Salmo 2	89,46737
Pig Crono 3	89,06832	Pig Coli 3	82,93077	Soy List 3	DNA was too fragmented	Pig Salmo 3	89,4086
Soy Crono 1	87,13998	Soy Coli 1	88,05545	Peas List 1	DNA was too fragmented	Soy Salmo 1	87,93873
Soy Crono 2	88,46208	Soy Coli 2	91,74899	Peas List 2	DNA was too fragmented	Soy Salmo 2	89,06861
Soy Crono 3	88,4132	Soy Coli 3	93,32131	Peas List 3	DNA was too fragmented	Soy Salmo 3	88,81941

Supplementary Table S6. Relative abundances of the detected pathogenic bacterial taxa using metagenomic sequencing in all included meat and meat analogues where a cocktail of the four tested bacteria was inoculated into the matrix and incubated over time. 1, 2 and 3 represent the 3 days of incubation, day 1, day 2 and day 3.

	C. sakazakii	*E. coli*	*L. monocytogenes*	*S.* Typhi
Beef Mix 1	37,33244	4,92845	2,05032	41,67876
Beef Mix 2	28,36945	1,88599	0,34616	53,35242
Beef Mix 3	25,02477	1,4614	0,3375	55,63213
Chicken mix 1	16,07143	9,19263	0,40249	40,41137
Chicken Mix 2	20,70064	0,1614	0,1833	55,94276
Chicken mix 3	21,02403	0,14638	0,31021	56,65993
Peas Mix 1	43,17743	7,05854	2,5974	36,20287
Peas Mix 2	40,2535	6,46692	1,40915	41,49422
Peas Mix 3	42,50316	7,17402	2,16702	38,37138
Pig Mix 1	30,19369	5,49055	1,11744	52,31025
Pig Mix 2	23,54396	5,57115	0,51391	59,93487
Pig Mix 3	19,69264	5,42421	0,43858	63,73938
Soy Mix 1	48,14465	6,64891	1,044	31,95878
Soy Mix 2	52,90847	5,49428	0,42682	30,64225
Soy Mix 3	52,25041	6,2094	0,71771	29,5788

Supplementary Table S7. Overview of both pathogen detection methods from all included meat and meat analogues, and all tested bacterial taxa. Culturomics are in CFU/100 µL and metagenomics are in relative abundances (%). The metagenomics output are from the samples that were spiked with the bacterial cocktail as described in the main text. 1, 2 and 3 represent the 3 days of incubation, day 1, day 2 and day 3.

	Chicken		Beef		Pig		Soy		Peas	
	culturomic	metagenomics	culturomic	metagenomics	culturomic	metagenomics	culturomic	metagenomics	culturomic	metagenomics
E. coli 1	2	9,19263	30	4,92845	86	5,49055	9,3	6,64891	3,5	7,05854
E. coli 2	1	0,1614	64	1,88599	110	5,57115	25	5,49428	13	6,46692
E. coli 3	4	0,14638	260	1,4614	98	5,42421	15	6,2094	16	7,17402
Listeria 1	110	0,40249	140	2,05032	200	1,11744	130	1,044	86	2,5974
Listeria 2	38	0,1833	74	0,34616	30	0,51391	120	0,42682	40	1,40915
Listeria 3	37	0,31021	37	0,3375	8	0,43858	120	0,71771	67	2,16702
Cronobacter 1	470	16,07143	450	37,33244	150	30,19369	180	48,14465	130	43,17743
Cronobacter 2	380	20,70064	220	28,36945	180	23,54396	100	52,90847	91	40,2535
Cronobacter 3	210	21,02403	200	25,02477	310	19,69264	56	52,25041	100	42,50316
Salmonella 1	780	40,41137	390	41,67876	490	52,31025	290	31,95878	180	36,20287
Salmonella 2	400	55,94276	420	53,35242	440	59,93487	270	30,64225	140	41,49422
Salmonella 3	140	56,65993	310	55,63213	320	63,73938	190	29,5788	130	38,37138

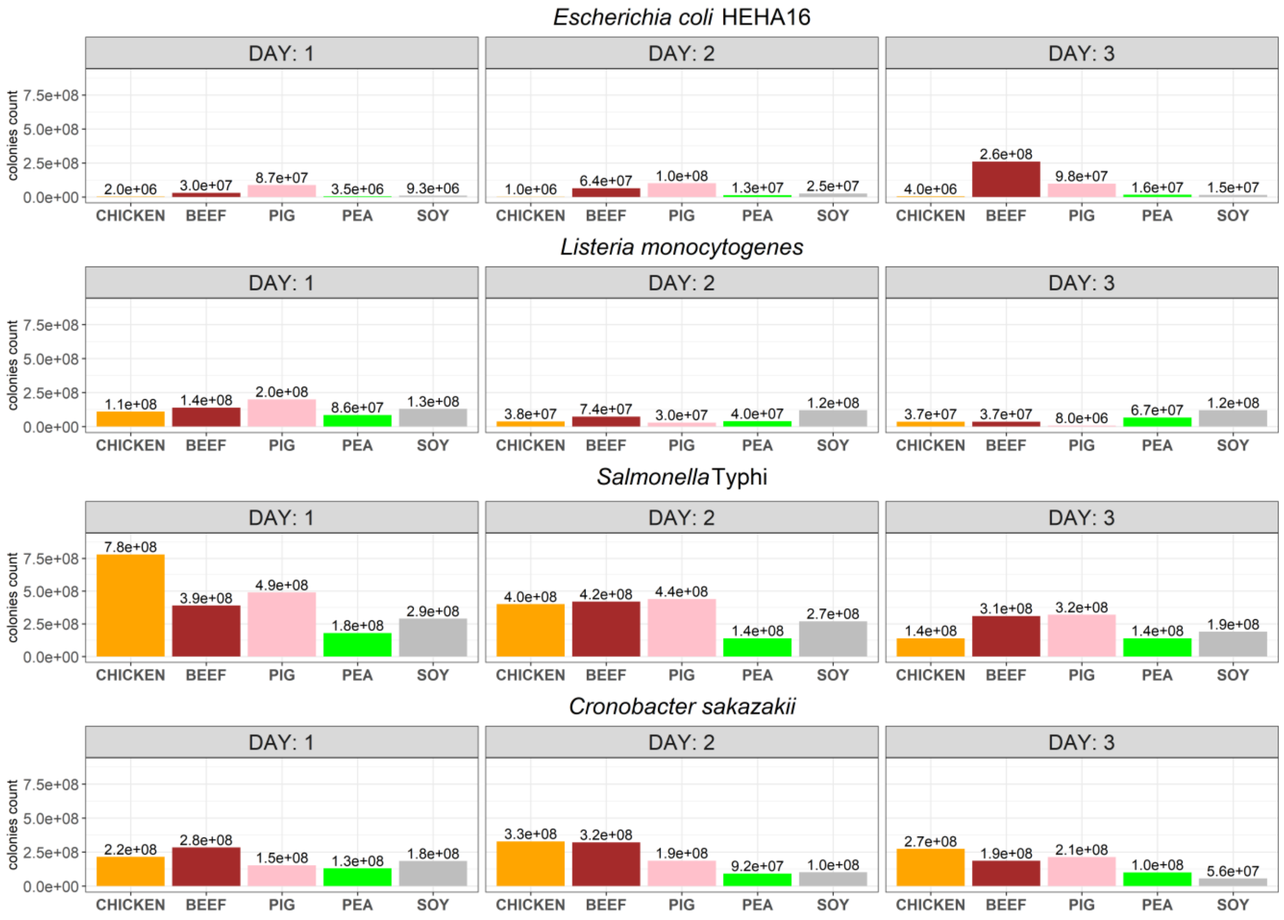

Supplementary Figure S1. Foodborne bacterial pathogen growth and survival over time in four meat and meat analogues using culture-dependent approach. Each bar represents the counted CFUs after the initial artificial inoculation date (day 0). Each number was adjusted to represent only the bacteria growth after adding the starting bacterial inoculum, and all CFU counts are adjusted to CFU/100 µL volumes of the initial matrix-bacterial mix to be able to compare the outputs.

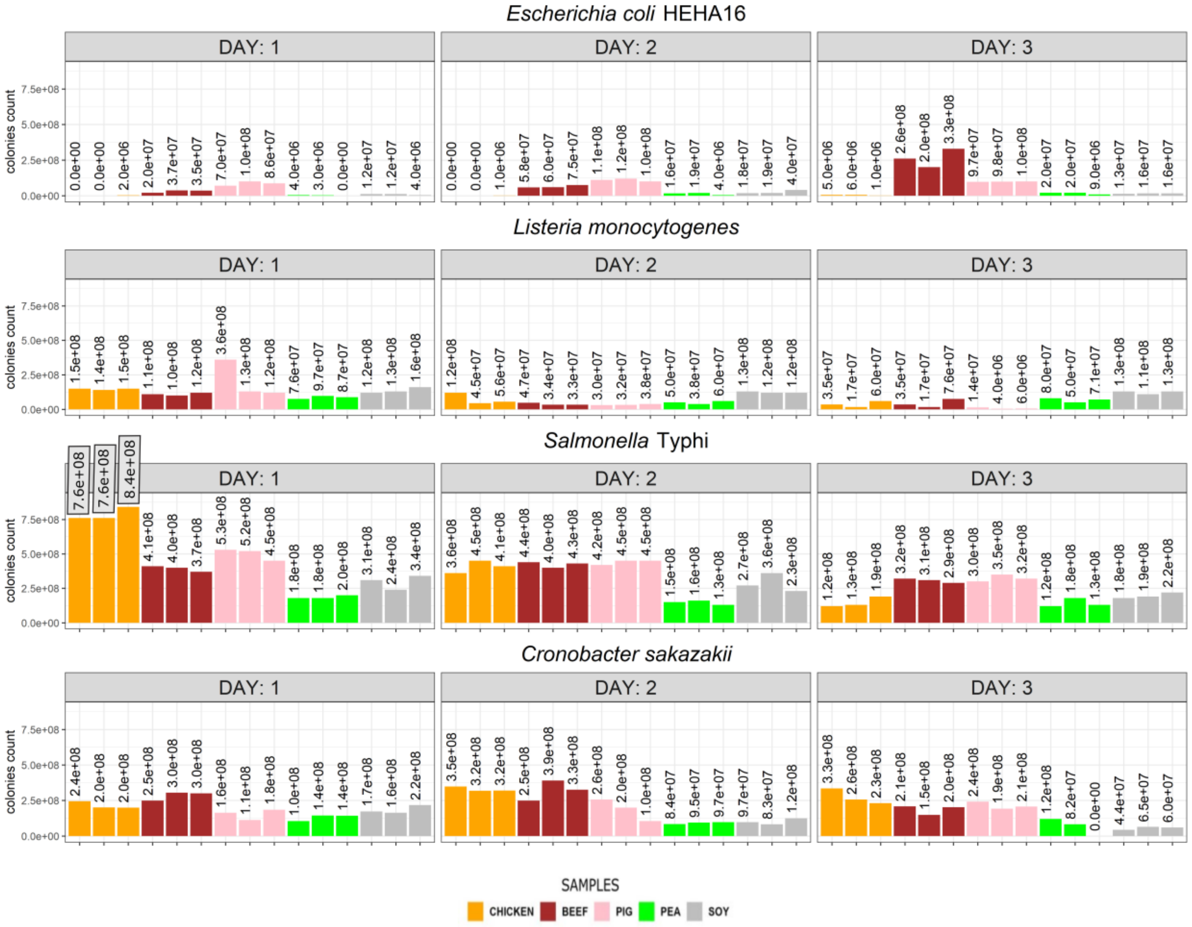

Supplementary Figure S2. Foodborne bacterial pathogen growth and survival over time in four meat and meat analogues using culture-dependent approach. Each bar represents the counted CFUs after the initial artificial inoculation date (day 0). Each number was adjusted to represent only the bacteria growth after adding the starting bacterial inoculum, and all CFU counts are adjusted to CFU/100 μL volumes of the initial matrix-bacterial mix to be able to compare the outputs.

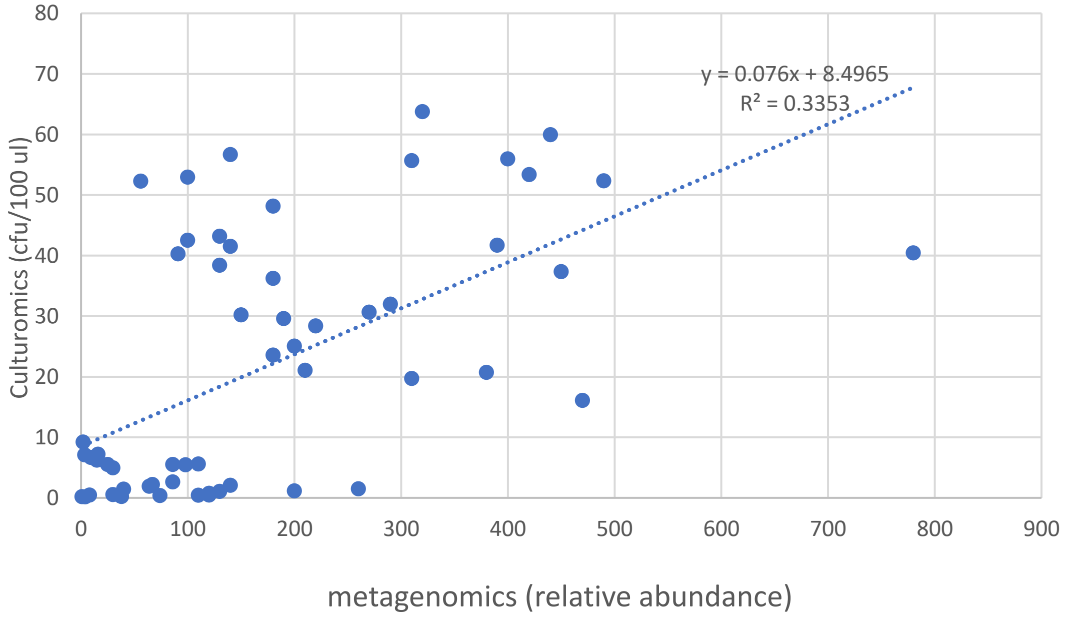

Supplementary Figure S3. Correlation between the detection levels of the foodborne pathogens using both culture-dependent methods (culturomics) and next generation metagenomic sequencing. With trend line and correlation R^2 values.
